# Remote Monitoring of Physiology in People Living With Dementia: An Observational Cohort Study

**DOI:** 10.2196/43777

**Published:** 2023-03-09

**Authors:** Michael C B David, Magdalena Kolanko, Martina Del Giovane, Helen Lai, Jessica True, Emily Beal, Lucia M Li, Ramin Nilforooshan, Payam Barnaghi, Paresh A Malhotra, Helen Rostill, David Wingfield, Danielle Wilson, Sarah Daniels, David J Sharp, Gregory Scott

**Affiliations:** 1 UK Dementia Research Institute Care Research and Technology Centre Imperial College London London United Kingdom; 2 Imperial College London, Brain Sciences South Kensington London United Kingdom; 3 Surrey and Borders Partnership NHS Foundation Trust Leatherhead Surrey United Kingdom

**Keywords:** dementia, remote monitoring, physiology, Internet of Things, alerts, monitoring, technology, detection, blood pressure, support, feasibility, system, quality of life

## Abstract

**Background:**

Internet of Things (IoT) technology enables physiological measurements to be recorded at home from people living with dementia and monitored remotely. However, measurements from people with dementia in this context have not been previously studied. We report on the distribution of physiological measurements from 82 people with dementia over approximately 2 years.

**Objective:**

Our objective was to characterize the physiology of people with dementia when measured in the context of their own homes. We also wanted to explore the possible use of an alerts-based system for detecting health deterioration and discuss the potential applications and limitations of this kind of system.

**Methods:**

We performed a longitudinal community-based cohort study of people with dementia using “Minder,” our IoT remote monitoring platform. All people with dementia received a blood pressure machine for systolic and diastolic blood pressure, a pulse oximeter measuring oxygen saturation and heart rate, body weight scales, and a thermometer, and were asked to use each device once a day at any time. Timings, distributions, and abnormalities in measurements were examined, including the rate of significant abnormalities (“alerts”) defined by various standardized criteria. We used our own study criteria for alerts and compared them with the National Early Warning Score 2 criteria.

**Results:**

A total of 82 people with dementia, with a mean age of 80.4 (SD 7.8) years, recorded 147,203 measurements over 958,000 participant-hours. The median percentage of days when any participant took any measurements (ie, any device) was 56.2% (IQR 33.2%-83.7%, range 2.3%-100%). Reassuringly, engagement of people with dementia with the system did not wane with time, reflected in there being no change in the weekly number of measurements with respect to time (1-sample *t*-test on slopes of linear fit, *P*=.45). A total of 45% of people with dementia met criteria for hypertension. People with dementia with *α*-synuclein–related dementia had lower systolic blood pressure; 30% had clinically significant weight loss. Depending on the criteria used, 3.03%-9.46% of measurements generated alerts, at 0.066-0.233 per day per person with dementia. We also report 4 case studies, highlighting the potential benefits and challenges of remote physiological monitoring in people with dementia. These include case studies of people with dementia developing acute infections and one of a person with dementia developing symptomatic bradycardia while taking donepezil.

**Conclusions:**

We present findings from a study of the physiology of people with dementia recorded remotely on a large scale. People with dementia and their carers showed acceptable compliance throughout, supporting the feasibility of the system. Our findings inform the development of technologies, care pathways, and policies for IoT-based remote monitoring. We show how IoT-based monitoring could improve the management of acute and chronic comorbidities in this clinically vulnerable group. Future randomized trials are required to establish if a system like this has measurable long-term benefits on health and quality of life outcomes.

## Introduction

Dementia engenders a significant burden to patients, carers, and health care services. In the United Kingdom (UK), there are an estimated 850,000 people with dementia, a number expected to rise to over 2 million by 2051 [1]. Dementia care costs the National Health Service approximately £23 (US $27.88) billion per year. There is a pressing need for interventions that reduce the burden on health care services and carers.

In addition to cognitive and behavioral symptoms, dementia is commonly associated with long-term comorbidities, including hypertension, diabetes, malnutrition or unintentional weight loss, and heart disease [2-5]. Many such comorbidities are adverse factors in its progression [2,3,6-10] but are underrecognized and undertreated [11,12]. People with dementia are also at increased risk of hospital admission especially for infections and falls [3,13-15]. People with dementia are more likely to die during admissions, and over a third who go into hospital from home are discharged to a care home [16,17]. Abnormal physiological measurements are more common in people with dementia because of autonomic dysfunction, comorbidities, medication side effects, and acute illnesses [18-21]. People with dementia are also at increased risk of frailty [22]. The interaction between frailty and acute illnesses confers a multiplicative risk for significant morbidity and mortality [23]. A combination of poor premorbid function with atypical presentations and a reduced ability to describe and communicate symptoms drives the increased risk [24]. Recognizing and treating illnesses early leads to better outcomes, especially in the elderly [25].

Using “Internet of Things” (IoT) technology, physiological measurements can be recorded at home and transmitted automatically to caregivers [26]. Such technology can improve the monitoring and treatment of comorbidities and detect developing acute illness [26]. Higher temporal frequency of measurements in a more “naturalistic” setting can potentially provide more accurate, granular data on patients’ health. It also reduces the need for patients with reduced mobility to travel to access care. Therefore, IoT could improve the health and quality of life of people with dementia and reduce the burden on services [26]. Furthermore, by involving people with dementia in their own care, we can maximize empowerment regarding their own health [27]. This is vital for effective ethical care and can be in part enabled through technology [28]. There are also likely benefits to carers who can be involved directly in the ongoing assessments while having increased confidence to leave the people with dementia alone.

There is considerable interest from health and social care policy makers in systems that enable remote monitoring of physiological parameters in community-dwelling people with dementia [29,30], especially given the COVID-19 pandemic [31]. However, in-home monitoring creates new challenges for clinical practice. Guidelines for the frequency of remote measurements (eg, daily, weekly), definitions of clinically significant abnormalities (eg, the threshold heart rate [HR] of clinically relevant tachycardia), and algorithms for best-practice care are not established, unlike in the inpatient setting. The UK-wide National Early Warning Score 2 (NEWS, Table S1 in Multimedia Appendix 1) defines illness severity for hospitalized patients using a score aggregated from 6 physiological domains, where a higher subscore means the parameter is further from normal [32]. However, it is unclear whether NEWS, validated for inpatients, is suitable in the home setting. Also, little is known about the distribution of physiological measurements in people with dementia recorded in the community, which is crucial to establish when designing a system to detect abnormalities.

We are unaware of any published data from long-term studies employing IoT devices for physiological monitoring in the homes of people with dementia.

We have developed an IoT platform, “Minder,” that enables physiological measurements to be recorded at home and monitored remotely [33]. Here, we carried out an analysis of the physiology (HR, systolic blood pressure [SBP] and diastolic blood pressure [DBP], oxygen saturation, and body weight) recorded by a group of people with dementia at home. We report on the effectiveness of our Minder system, designed to detect abnormal measurements (“alerts”) and direct a clinical monitoring team. We also retrospectively applied the NEWS criteria to the data as a comparator. Finally, we present case studies highlighting the potential benefits of remotely monitoring people with dementia.

Our aims are to (1) characterize the physiology of people with dementia in their home setting, (2) test whether our system is sensitive to comorbidities and dementia subtypes, and (3) test how well NEWS-style alerts systems translate to community measurements.

## Methods

### Study Design, Participants, and Recruitment

We are conducting an ongoing longitudinal community-based cohort study of people with dementia living at home using Minder, passive infrared sensing, and data analytics to enable remote health care monitoring [33]. Patients with an existing clinical diagnosis of dementia of any cause were recruited from primary care, adult social care services, and memory clinics across Surrey and Borders Partnership NHS Foundation Trust and Hammersmith and Fulham Partnership. People with dementia were enrolled with an associated “study partner,” defined as “a relative or friend who has known the people with dementia for at least 6 months.” A distinction was not made between “study partner” and “carer”; however, the average number of hours the study partners spent caring for their respective people with dementia was 5.4 (range 1-8) for the 40 study partners for whom we have this data. Full inclusion and exclusion criteria are listed in “Methods” in Multimedia Appendix 2. Owing to the developmental and exploratory nature of the ongoing study, the number of participants was not predetermined by a power calculation. The study is reported according to the STROBE (Strengthening the Reporting of Observational Studies in Epidemiology) statement (Multimedia Appendix 3), guidelines for reporting observational studies [34].

### Ethics Approval

The study was approved by the Health Research Authority’s London-Surrey Borders Research Ethics Committee (19/LO/0102). All people with dementia and study partners provided written informed consent for participation and for their data to be included in publications.

### Study Procedures and Physiological Measurements

The study protocol and devices used were based on a previous trial [26]. That trial was co-designed with 20 people with dementia, carers, health care and social workers, and academics, who designed the system to be appropriate for use in people with dementia, in addition to data from an Alzheimer's Society survey on technology-enhanced care. The system was first tested in a laboratory setting and home mock-up scenario before being deployed in participants’ homes.

At baseline, people with dementia and their study partners completed demographics questionnaires and people with dementia completed the Standardized Mini-Mental State Examination (SMMSE). All people with dementia received up to 4 IoT medical devices to record 6 physiological measurements: a blood pressure machine for SBP and DBP, a pulse oximeter measuring oxygen saturation and HR, and body weight scales (provided by iHealth), and a thermometer (provided by Withings), and were asked to use each device once a day at any time. Measurements recorded by each device, annotated with a datetime stamp, were automatically transmitted immediately to a centralized secure server.

### Study Oversight and Minder Alert Criteria

All people with dementia and study partners provided written informed consent. To oversee the study, a monitoring team was established, operating from 9 AM to 5 PM daily, to respond to clinical or technical alerts. The monitoring team was supervised by a consultant psychiatrist, 2 consultant neurologists, a general practitioner, and an occupational therapist. The team had near real-time access to the physiological measurements via a clinical dashboard, which additionally alerted staff when observations met standardized criteria devised by the study team (Table S2 in Multimedia Appendix 4). For any alert, the monitoring team would follow a predefined flowchart to investigate the abnormality, beginning by attempting to contact the people with dementia or study partner (Figures S1-S4 in Multimedia Appendices 5-8, respectively).

### Statistical Analyses

All analyses were performed in MATLAB [35]. Data distributions were assessed for normality. Mean and SD are reported for Gaussian data (as per the Shapiro-Wilk test), whereas median and IQR are used for non-Gaussian data. We calculated descriptive statistics for baseline demographics and SMMSE, and grouped these data by dementia subtype, defined as Alzheimer disease (AD), vascular dementia (VD), and *α*-synuclein–associated dementias (ASyn), that is, combining participants with Parkinson disease dementia and Lewy body dementia.

We summarized the 24-hour timing of measurements by grouping timestamps into hourly bins. We calculated the overall frequency of recordings by dividing the number of days where a participant took at least 1 complete set (ie, all devices available) of measurements by the number of days of observation; we also counted the days when any measurement was taken. To examine whether the frequency of measurements changed over time, for each participant, we counted the number of measurements per week; fit a linear model to this time series; and, at the group level, tested whether the slopes of these fits were significantly different to zero (1-sample *t* test).

For subsequent analyses, measurement values were excluded as outliers if values were greater than 4 SD from the mean for that participant. For blood pressure, we calculated the proportion of people with dementia whose mean values met clinical criteria for hypertension (SBP/DBP ≥135/85 mg) [36] or hypotension (SBP/DBP ≤90/60 mm Hg) [37].

We charted the body weight of people with dementia over time, using a sliding window average of 5 values. To identify clinically significant weight loss (or gain) [38] in participants with at least 6 months of data, we identified averaged weights that were >5% different, in either direction, from the most recent value recorded at least 6 months previously.

We refer here to 2 different sets of alert thresholds: our own Minder thresholds and the established NEWS thresholds (Table S1 in Multimedia Appendix 1 and Table S2 in Multimedia Appendix 4). The Minder thresholds were used in real time during the study, to alert the monitoring team, whereas the NEWS thresholds were applied retrospectively to the data for comparison. When applying the subscore thresholds of the NEWS [32] to the data, we first removed any repeat measurements recorded within 60 seconds. For each domain within the NEWS criteria, a normal value is scored 0, but 1-3 when values meet predetermined criteria for abnormality (Table S1 in Multimedia Appendix 1), and these subscores are aggregated into a single NEWS score [32]. For each participant, we calculated the number of individual measurements that triggered Minder alerts and NEWS thresholds of 1+ (less abnormal) and 2+ (more abnormal). We then summarized the overall “burden” of alerts per day per participant.

We used the Spearman rank to test for correlations between physiological summary measures and baseline SMMSE scores and 1-way ANOVA to test for differences between the dementia subtypes (AD, VD, ASyn), with post hoc Tukey tests. Owing to the exploratory nature of the analysis, we did not correct for multiple comparisons.

### Case Studies

Four case studies, each based on a snapshot from an individual person with dementia, have been identified. These are included for the purpose of demonstrating the use of this monitoring system for detecting acute clinical events as well as chronic changes in physiological measures over time.

## Results

### Participant Characteristics, Dementia Subtypes, and Analysis Period

**Figure 1:**
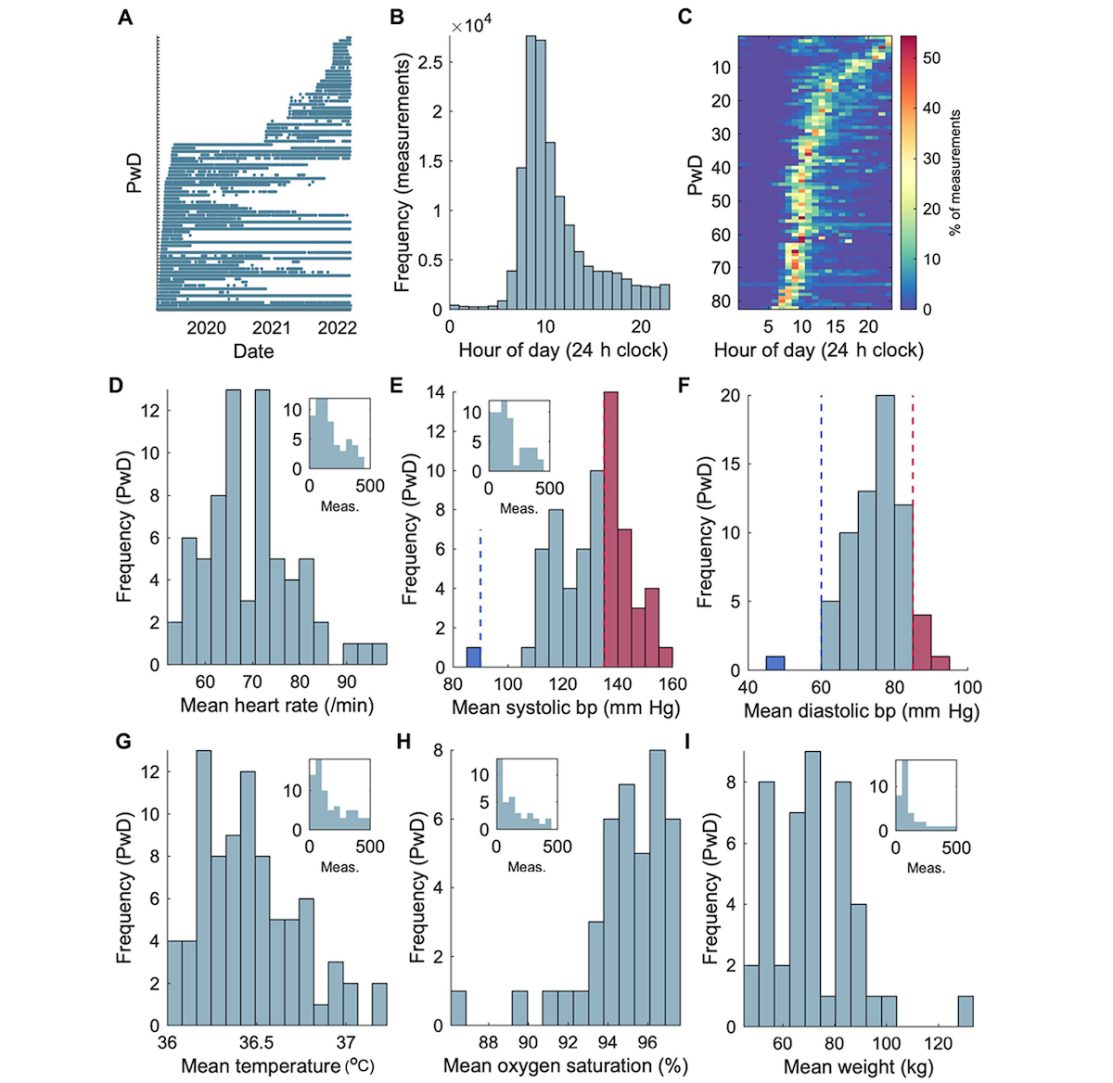
Timings and mean values of in-home measurements taken by PwD. (A) Each horizontal line represents a participant, and each dot represents a recording. Rows have been ordered by date of first observation. (B) Count of measurements by each hour of the day, across the cohort. (C) Timing of each participant’s measurements group per hour of the day, expressed as a percentage of that participant’s total measurements. Rows have been ordered by the hour of maximum percentage of most observations (earliest to latest; a different ordering to panel A). (D) Within-participant mean heart rate (beats per minute, n=66 participants); only participants with more than 7 days’ data are included for D-G. The inset histograms show the number of measurements (x-axis) recorded by each participant (y-axis) for the domain. (E) Systolic blood pressure (bp, mm Hg, n=66). Coloring of bars indicate cutoffs for clinical diagnoses of hypertension (≥135 mm Hg, red) and hypotension (≤90 mm Hg, blue). (F) Diastolic blood pressure (bp, mm Hg, n=66). Coloring of bars indicate cutoffs for hypertension (≥85 mm Hg, red) and hypotension (blue, ≤60 mm Hg). (G-I) Mean calculated from all measurements for each individual of body temperature (°C, n=82), oxygen saturation (%, n=40), body weight (kg, n=44). bp: blood pressure; PwD: people with dementia.

Data from 82 people with dementia were analyzed, with a mean age of 80.4 (SD 7.8, range 60.5-96.4) years at study entry; 36 (44%) were women. Table 1 shows baseline participant characteristics including SMMSE scores (mean 23.0, SD 4.2), grouped by dementia subtypes—AD, VD, and ASyn. The medical history of people with dementia was accessed via their general practitioner (GP) records on enrollment: 1 had type 1 diabetes mellitus and 2 had type 2 diabetes mellitus. Although 18 records stated that they had a diagnosis of essential hypertension, 29 were on at least 1 medication with antihypertensive action (calcium channel blockers, angiotensin-converting enzyme inhibitors, *β*-blockers, *α*-blocker, angiotensin receptor blockers, and diuretics). There was a significant difference in age between dementia subtypes (1-way ANOVA *F*_2,79_=5.346, *P*=.007), with participants with AD older than those with ASyn (post hoc Tukey test, *P*=.004). There was no difference in baseline SMMSE scores between dementia subtypes (*F*_2,78_=2.324, *P*=.11). Recruitment to the study was ongoing throughout, and thus those included commenced the study at different points in the analysis period: April 1, 2019, to March 14, 2022 (1078 days; Figure 1A). The median number of days of observations per patient, defined as the days between the first and last recorded measurement, was 432.5 (IQR 164.9-764.0, range 15.8-1077.1) days, a total of 957,861 participant-hours. A total of 37 participants withdrew from the study during the analysis period, including 5 (6%) who died. The most frequent reason for withdrawal was people with dementia moving to a care home. Full details of the withdrawals are reported in Table S3 in Multimedia Appendix 9. Given the nature of the population in this observational study, it was expected that a significant proportion would withdraw or pass away, as their condition progressed.

**Table 1:** Baseline participant characteristics, grouped by dementia subtype (N=82).^a^

Variable		Overall	Dementia type			
			AD^b^ (n=68)		VD^c^ (n=5)	ASyn^d^ (n=9)
Age (years), mean (SD)		80.4 (7.8)	81.5 (7.5)		80.1 (10.2)	72.8 (4.8)
**Sex, n (%)**						
	Female	36 (44)		33 (49)	1 (20)	2 (22)
	Male	42 (56)		35 (52)	4 (80)	7 (78)
	Other	0 (0)		0 (0)	0 (0)	0 (0)
White ethnicity, n (%)		77 (94)	61 (90)		4 (80)	8 (89)
SMMSE^e^ (out of 30), mean (SD)		23.0 (4.2)	22.8 (4.1)		26.8 (2.9)	22.3 (4.7)
**Education, n (%)**						
	Up to 16	35 (45)		28 (44)	2 (40)	5 (56)
	Up to 18	14 (18)		14 (22)	0 (0)	0 (0)
	Vocational^f^	8 (10)		5 (8)	1 (20)	2 (22)
	University degree	21 (27)		17 (27)	2 (40)	2 (22)

^a^Data from questionnaires completed by participants on enrolment.

^b^AD: Alzheimer disease.

^c^VD: vascular dementia.

^d^ASyn: *α*-synuclein–associated disorders (Parkinson disease dementia and Lewy body dementia combine).

^e^SMMSE: Standardized Mini-Mental State Examination.

^f^Where data are incomplete, percentage calculated from available data only. Vocational education refers to higher education course (above the age of 16 years) toward a specific vocation, rather than 16- to 18-year-old schooling or university degree.

### Number, Timing, and Frequency of Physiological Measurements

There were 147,203 individual measurements recorded among the 82 participants. Measurements were most often recorded in the morning, with 8-9 AM the most frequent hour (Figure 1B), but there was wide between- and within-participant variability (Figure 1C). We defined participants’ frequency of physiological measurement recording in 2 ways. The median proportion of days of observation during which any participant took at least 1 full set of measurements was 13.9% (IQR 1.2%-33.1%, range 0%-61.0%). The median percentage of days when people with dementia took at least 1 measurement using any device was 56.2% (IQR 33.2%-83.7%, range 2.3%-100%) and did not differ between dementia subtypes (*F*_2,79_=0.944, *P*=.91). By either definition, there was no correlation between frequency and SMMSE (Spearman *ρ P*=.17 and *P*=.095, respectively). We also examined whether measurement frequency changed over the study, that is, that might reflect study fatigue or difficulties with devices. There was no change in the weekly number of measurements in people with dementia with respect to time (1-sample *t* test on slopes of linear fit, *P*=.45). There was also no correlation between any change in frequency (fitted slopes) and SMMSE (*P*=.34).

### Values of Physiological Measurements and Prevalence of Hypertension

Figure 1D-I shows for each measurement domain the distributions of participants’ mean values recorded during the study for people with dementia with more than 7 days of measurements. Grand means, calculated as the mean of all the within-subject means, were as follows: group mean HR 69.6 (SD 9.4, range 53.5-97.4) bpm, mean SBP 131.7 (SD 14.1, range 85.0-165.9) mm Hg, mean DBP 74.9 (SD 7.5, range 47.7-90.0) mm Hg, median temperature 36.4 (IQR 36.2-36.6, range 36.0-37.2) °C, median oxygen saturation 95.2% (IQR 94.4%-96.5%, range 86.7%-97.5%), median body weight 71.4 (IQR 61.8-83.1, range 48.7-132.0) kg. Using typical clinical criteria [36,37], 45.4% of people with dementia with available data had hypertension (within-subject mean either SBP/DBP ≥135/85 mg), and 1 had hypotension (within-subject mean either SBP/DBP ≤90/60 mm Hg; Figure 1D and E**)**. Figure S5A-F in Multimedia Appendix 10 shows the distributions of within-participant SDs of values.

### Physiological Measurements Between Dementia Subtypes

**Figure 2:**
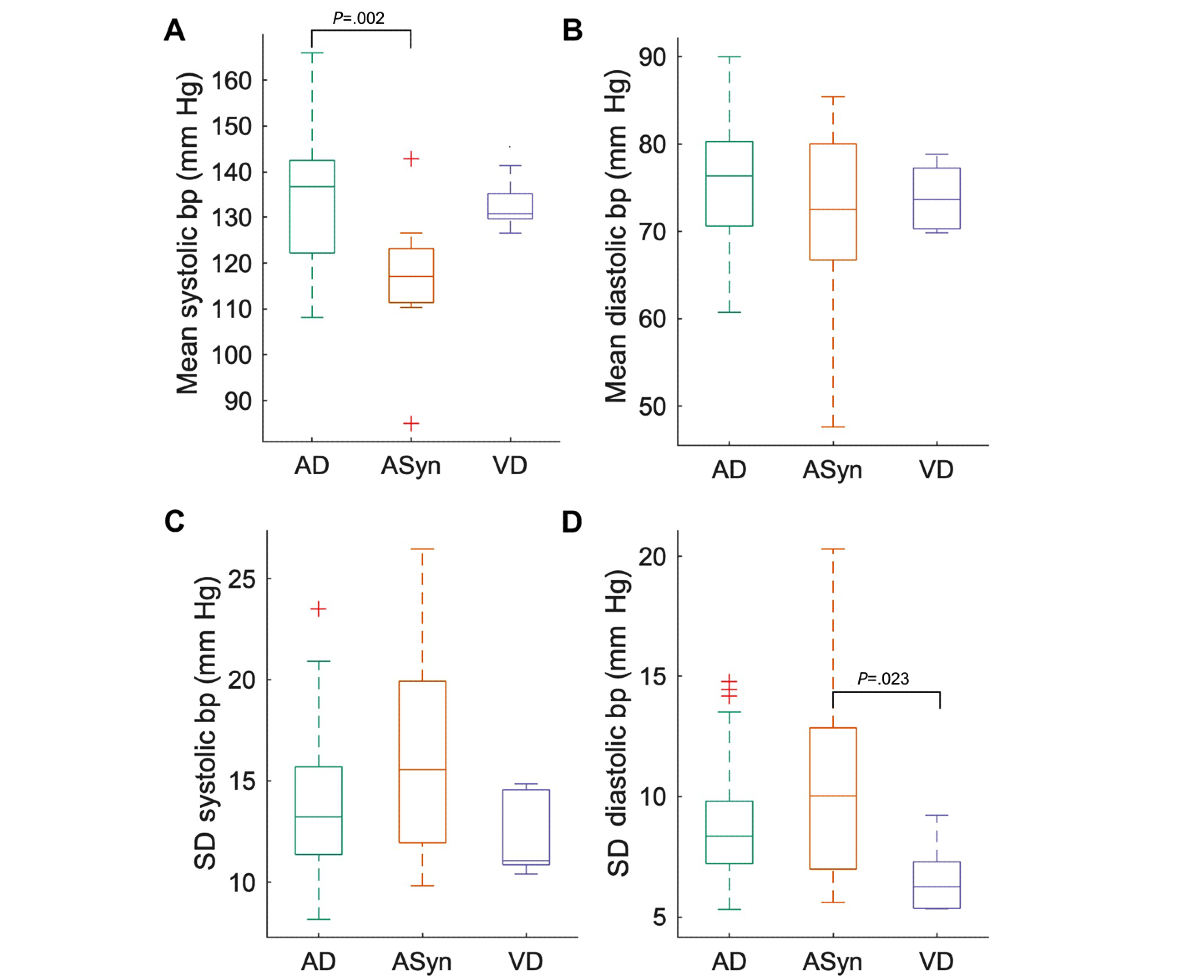
Within-participant means and SDs of physiological measurements in people with dementia, grouped by dementia subtypes. Only participants with more than 7 days of data are included. Results of post hoc Tukey test are shown where significant (*P*<.05). (A) Mean systolic blood pressure (SBP, mm Hg). (B) Mean diastolic blood pressure (DBP, mm Hg). (C) SD of SBP (mm Hg). (D) SD of DBP (mm Hg). AD: Alzheimer disease; ASyn: *α*-synuclein–associated disorders; bp: blood pressure; VD: vascular dementia.

We grouped the within-participant means and SDs by dementia subtypes (Figure 2A-D for SBP/DBP; see Figure S6 in Multimedia Appendix 11 for other domains). There was a significant difference across subtypes in mean SBP (*F*_2,63_=6.203, *P*=.003), SD of DBP (*F*_2,63_=3.790, *P*=.03, post hoc Tukey test results shown in Figure 2), and SD of oxygen saturation (*F*_2,37_=6.317, *P*=.004), with ASyn higher than AD participants (post hoc Tukey test, *P*=.005) and VD participants (post hoc Tukey test, *P*=.01).

### Body Weight and Prevalence of Clinically Significant Weight Loss

**Figure 3:**
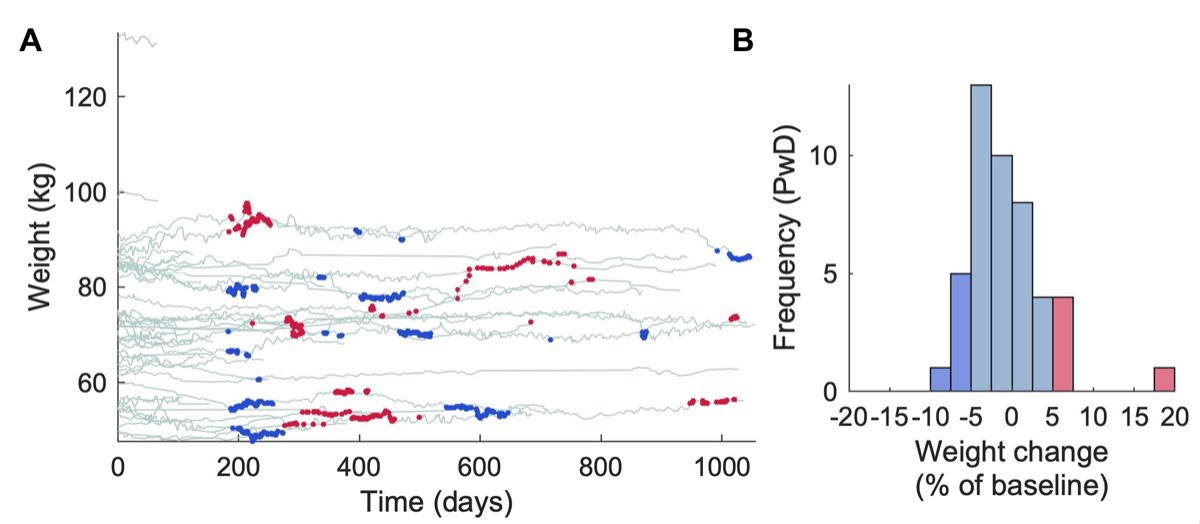
Body weight over time in PwD. (A) Participants’ body weight over time. Each line represents a different PwD, and each point on the line represents the sliding window average value of 5 body weights. The blue segments indicate values that were >5% less than values 6 months previously, suggesting clinically significant weight loss [38]; the red segments indicate >5% weight gain. (B) Participants’ final body weight (average of 5 measurements) is expressed as a percentage of their baseline weight; bins for >5% weight change are colored blue (loss) and red (gain). PwD: people with dementia.

Using the criteria of >5% change over 6 months [38] (Figure 3A), 13 (28%) participants who recorded weight measurements for at least 6 months had at least 1 period of weight loss during the study; 15 (33%) had weight gain. The median percentage change in body weight over the whole study was –1.6% (IQR –3.4% to 1.7%, range –8.4% to 17.6%; Figure 3B). There was no relationship between change in body weight and SMMSE (*P*=.54).

### Physiological Measurements Generating Alerts

To inform the development of remote monitoring services, we calculated the prevalence of abnormalities generating “alerts” according to several criteria.

**Figure 4:**
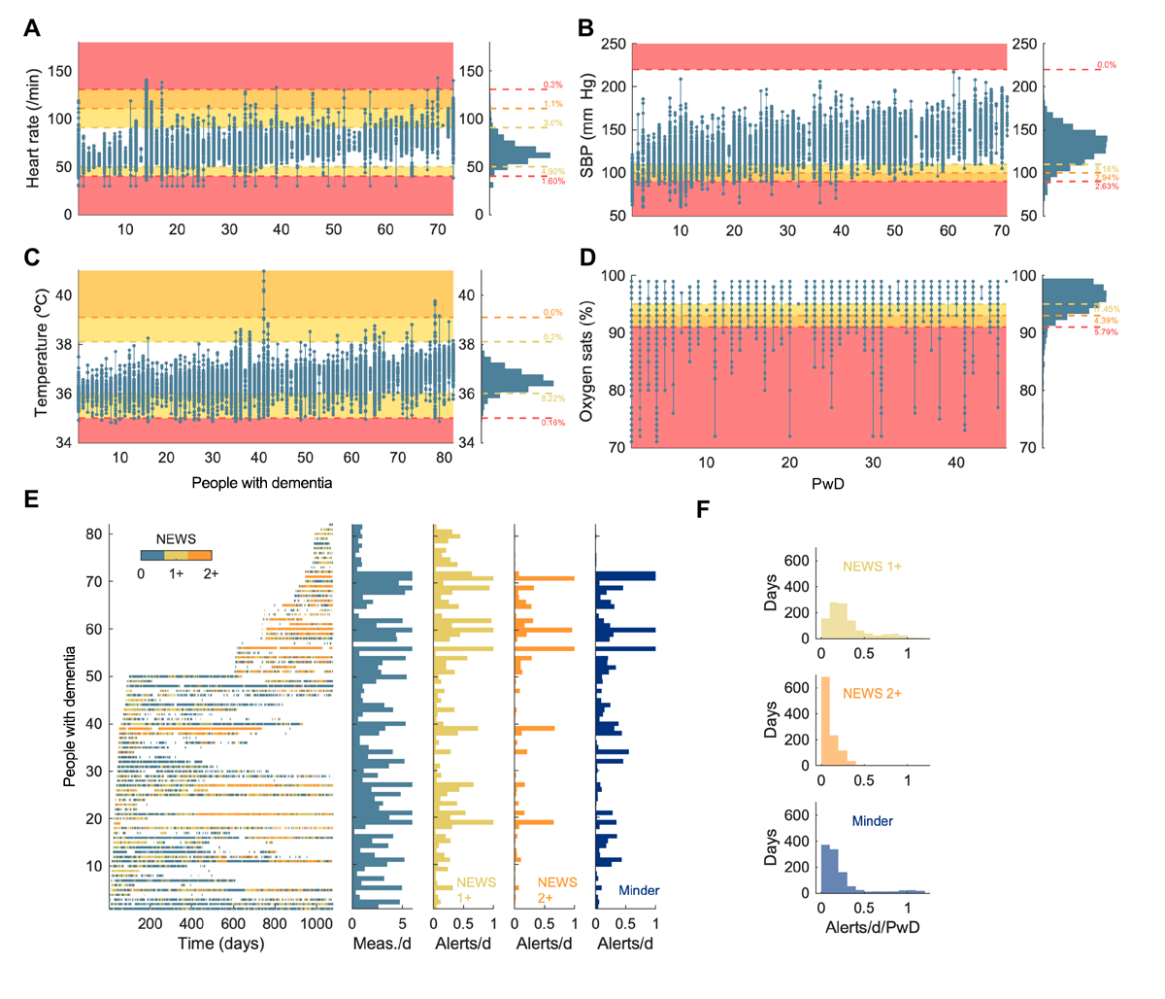
Frequency of physiological measurement alerts in people with dementia, by NEWS and Minder criteria. For each domain, each line with superimposed circles shows the minimum, maximum, and individual observations for each PwD, after first removing any measure recorded within 60 seconds of another in the same domain. The shaded areas correspond to a NEWS subscore of 1 (yellow), 2 (orange), or 3 (red). The histograms show the distribution of measurements across the group, in relation to the ranges for NEWS subscores, annotated with the percentage of measurements in each range. (A) Heart rate (beats per minute); (B) SBP (mm Hg); (C) temperature (°C); (D) oxygen saturation (%). Data were first filtered by removing any measure recorded within 60 seconds of another in the same domain. Alert for meeting the criteria of a NEWS subscore of 1 or more = NEWS 1+; alert for meeting the criteria of a NEWS subscore of 2 or more = NEWS 2+. (E) Data were labeled by a NEWS subscore of 0 (turquoise), 1+ (yellow), or 2+ (orange). Each horizontal row of circles (left) shows measurements for each participant, colored accordingly. Each subsequent column (left-right) shows the daily number of measurements (turquoise), and then the frequency of alerts using the criteria of NEWS 1+, NEWS 2+, and the Minder platform. (F) Histograms of the alerts per day per participant across the duration of the study, for criteria of NEWS 1+ (top), NEWS 2+ (middle), and the Minder platform (bottom). NEWS: National Early Warning Score 2; PwD: people with dementia; SBP: systolic blood pressure.

The prevalence of abnormalities recorded by people with dementia according to NEWS subscore criteria is shown in Figure 4A-D. In-home measurement domains were here treated independently, that is, not combined into a single NEWS score, because people with dementia did not necessarily record measures contemporaneously and because the full NEWS data, that is, respiratory rate and conscious level, were not captured. Instead, alerts could be generated for measurements in a single domain if they crossed either the 1+ or 2+ thresholds.

We retrospectively calculated the burden of alerts that would have been generated using NEWS subscore criteria, that is, the proportion and rate of measurements that exceeded different subscore thresholds (Figure 4). A total of 9.46% of all measurements would generate an alert for meeting the criteria of a NEWS subscore of 1 or more (1+), and 3.03% of measurements using a NEWS subscore of 2+. By comparison, the proportion using our Minder study criteria (Table S2 in Multimedia Appendix 4) was 7.88%. We summarized the alerts per participant per day to indicate the potential overall alert burden (Figure 4). The median frequency of alerts per day per participant was 0.233 (IQR 0.14-0.37; range 0-1.33) using NEWS 1+ and 0.066 (IQR 0.02-0.15; range 0-0.67) using NEWS 2+. Using our Minder criteria, the median frequency was 0.140 (IQR 0.07-0.25; range 0-1.17). There was no relationship between alerts per day, using either NEWS (1+ or 2+) or Minder criteria and SMMSE (*P*=.56, *P*=.54, *P*=.79, respectively) or dementia subtype (*P*=.92, *P*=.26, and *P*=.95).

### Case Studies

Here we report four case studies demonstrating the ability of the monitoring system to detect clinically relevant changes in physiological measurements. This provides evidence for the use of the system in picking up acute illness in a timely manner while also allowing clinicians to monitor chronic changes in individual patients.

**Figure 5:**
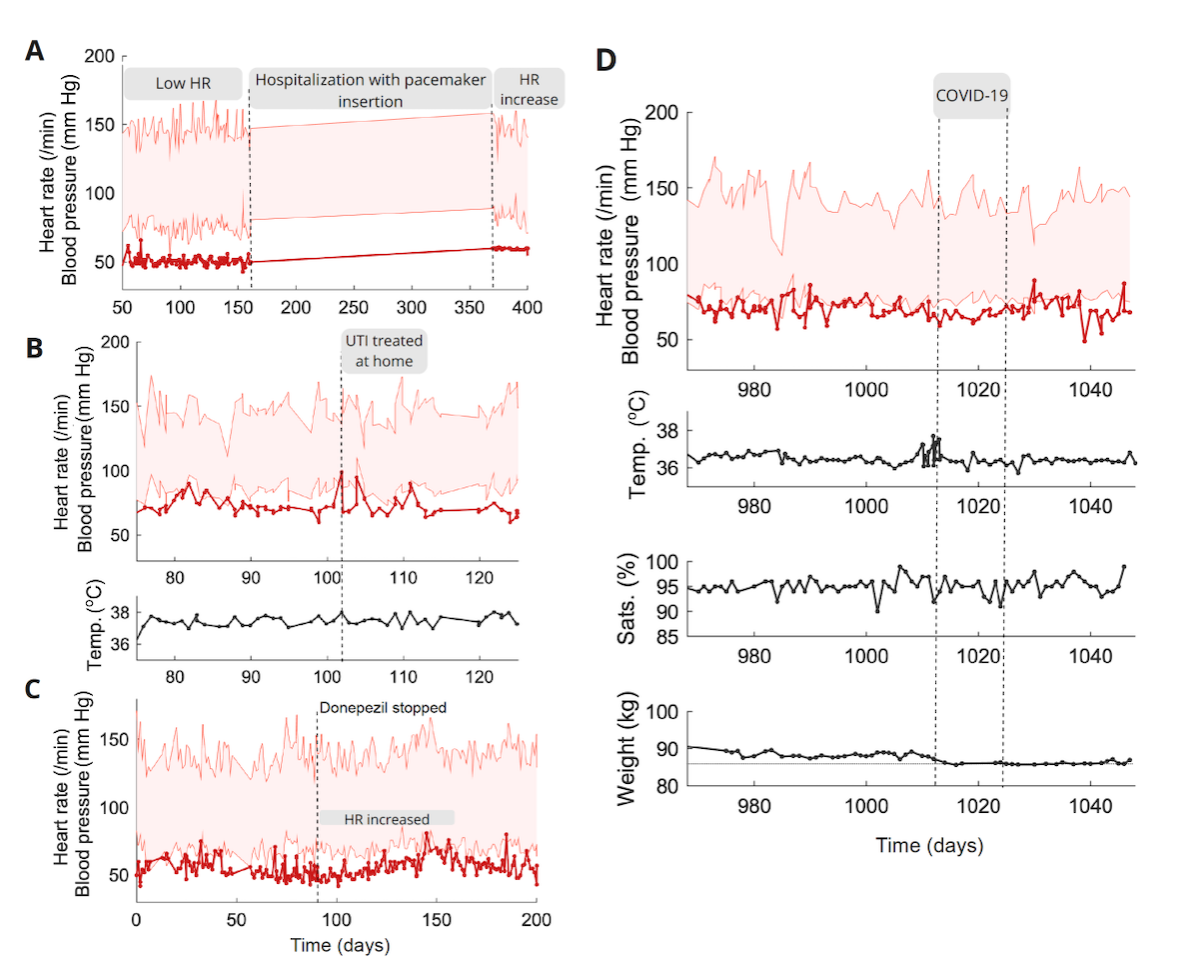
Case studies highlighting the potential benefits and challenges of remote physiological monitoring in people with dementia. Case studies are labeled according to descriptions in text. HR: heart rate; UTI: urinary tract infection.

1. Change in HR observations after pacemaker insertion: An 83-year-old man with AD was admitted to the hospital after a posterior myocardial infarction, resulting in a long gap in measurements (Figure 5A). After the infarction, he was persistently bradycardic (40-50 bpm) and had a permanent pacemaker fitted. Frequent HR readings of approximately 60 bpm followed the recommencement of home monitoring.
2. Physiological measurement abnormalities leading to urinary tract infection (UTI) diagnosis: An 81-year-old woman with AD had a temperature of 37.9 °C and HR of 102 bpm (Figure 5C). The monitoring team called the carer who relayed symptoms of a UTI. The carer was advised to call 111 (a public service for immediate health advice) and was seen at the hospital. A UTI was subsequently diagnosed, and treatment commenced at home.
3. Symptomatic bradycardia in a person with dementia on donepezil: A 78-year-old man with AD on donepezil recorded a series of low HR measurements over 3 weeks (lowest=44 bpm; Figure 5C), generating alerts for the monitoring team. The carer told the team that the person with dementia was more fatigued. He was advised to see the GP who switched donepezil, known to cause bradycardia and fatigue, for memantine. Our system showed a resultant increase in HR.
4. Remote physiological monitoring in a person with dementia with COVID-19 infection: An 81-year-old man with AD developed coryzal symptoms, a nonproductive cough, and a temperature of 38.37 °C (Figure 5E). Oxygen saturation remained >95%. Two days later, he was pyrexial again (38.57 °C) and tested positive for COVID-19 at home. The person with dementia was closely monitored with daily check-ins by the monitoring team. Ten days later, he tested negative. The person with dementia lost weight (89 to 85.7 kg after the infection). This was subsequently followed up by the GP.

## Discussion

### Study Scale

We have deployed IoT medical devices in the homes of a cohort of people with dementia, providing a rich data set of naturalistic physiological measurements. We believe that this is the first time the physiology of people with dementia has been recorded in this setting at such a scale and sustained period (approximately 150,000 measurements, approximately 1,000,000 participant-hours). We found a system of this nature to be realizable and effective in detecting acute and chronic physiological abnormalities.

### Principal Findings

We found that people with dementia recorded a full set of observations on 13.9% of days; however, at least 1 measurement was taken on more than half the study days, on average. Although the data were often incomplete, with measurement in some domains more likely to be recorded than others, there was no decline in compliance over time. Overall, concerns that people with dementia are unlikely to remember or be unwilling to have measurements taken in the community appear misplaced. However, these findings show that serial naturalistic data in people with dementia obtained remotely are likely to be patchy compared with what is possible in a nurse-led inpatient setting. Although there was no correlation between compliance and SMMSE in our data, it is possible that in more advanced dementia, there may be a reduction in compliance. However, although people with dementia with a lower SMMSE may be less likely to remember to take measurements, they are more likely to have a carer to support measurements.

Our system has provided an opportunity to describe the hitherto poorly understood behavior of physiological measures over extended periods in an older adult, cognitively impaired population, and to consider how physiology relates to comorbidity. On the basis of the data collected in this study, we detected a high prevalence of hypertension, an important factor in dementia development [8,9]. We also detected physiological differences between dementia subtypes, with lower and more variable blood pressure seen in people with dementia with ASyn, in keeping with the known associated autonomic dysfunction [39]. Our case studies provide further evidence that, at the individual level, remote monitoring can detect symptomatic bradycardia, acute infections, and medication side effects.

The relationship between cognitive decline and physical health is complex. For example, regarding blood pressure, both hypo- and hypertension have been implicated in the progression of cognitive decline [40]. Furthermore, because they have been largely excluded from previous randomized trials [41], the value of treating hypertension in older adult, cognitively impaired patients is not established. Body weight, as a marker of nutritional status, also has an important but complex interaction with dementia. We did not find an association with SMMSE, but it is likely that a longer time course would be required to detect one.

IoT-based platforms like Minder represent a new paradigm for clinical measurement, that is, large-scale, long-term, sporadic, patient-initiated, and remotely recorded. Definitions and care pathways for significant abnormalities in this context are not well established. These are important differences versus the established settings of primary care (infrequent, supervised, in-person measurements), secondary care (high-frequency in-person multimodal monitoring of acutely unwell patients), and ambulatory monitoring (devices worn continuously for several days). When we applied the NEWS criteria, validated for hospital use, we found that the median rate of alerts generated was 0.066 or 0.233 per day per participant, depending on the threshold used, which spanned the rate (0.114) from our own criteria. These findings provide an indication of the potential workload that would be placed on remote monitoring service (approximately 100 alerts per day per 1000 patients). There is, however, potential to improve the clinical use of such alerts, for example, by using personalization, whereby thresholds are set according to patients’ own historical data and constantly updated in response to their measurements.

### Future Directions

Our study highlights the benefits and risks of remote monitoring systems. With such systems, there is the potential to detect developing acute illness, facilitating early intervention, improving outcomes, and avoiding hospital admission [42,43]. Remote physiological monitoring of this kind could identify trends over weeks to months, relating to, for example, comorbidities like hypertension, malnutrition, and drug side effects. Both timescales are pertinent in people with dementia who, less able to recognize and communicate when they become acutely unwell, are more likely to develop comorbidities.

We are currently scaling up the size of the cohort in the study to 200 people with dementia in the form described here. There are further plans to provide a cut-down version of the system to a cohort of 1000. A key part of the ongoing work is ascertaining, which features are most informative, in order to design a system that is scalable.

It remains to be established the measurable benefit a system like this can have on long-term health outcomes and quality of life. A previous randomized trial did not point toward benefit, but this may well be due to a piecemeal approach [44]. In fact, successful implementation will depend on systematic work understanding how best to use technology in the home.

### Limitations

We have identified the following limitations to our IoT system and to the analysis presented. First, we were limited in our ability to reliably characterize and record every change in patients’ medical status and medication over the course of the study. This has implications for interpretation of the physiological findings. The association between changes in physiological observations and medication may be addressed using data linkage with the patient electronic clinical record—something we are exploring as the study develops. Second, we did not have a control group of age-matched healthy participants to provide comparisons for measurement compliance and physiological values. The normative values are for vital signs are well established, but not necessarily in the context of elderly people, in their own homes. Third, our analysis of compliance was limited as we could not discern which recordings were initiated by people with dementia versus in response to contact by the monitoring team. We therefore have likely overestimated the frequency with which people with dementia recorded measurements. However, the value in allowing people with dementia to record their vital signs remotely withstands, even if they have had to be prompted. Fourth, our alert rates are instead likely to be underestimates, because we excluded extreme outliers and duplicate values and did not evaluate abnormalities relating to DBP or body weight.

### Conclusions

There is growing interest in establishing remote monitoring within care services, amplified by the COVID-19 pandemic and calls for “hospital and home” initiatives [29,30]. We believe that remote monitoring technology can be transformative for the health and social care of people with dementia. Future research must demonstrate the clinical use of remote monitoring and address how such technologies are best integrated with existing care. Together, our findings inform the development of technologies, pathways, and policies for remote monitoring of people with dementia.

## Appendix

Multimedia Appendix 1 Threshold values for abnormal measurements in the National Early Warning System 2.

Multimedia Appendix 2 Methods: inclusion and exclusion criteria of study partners.

Multimedia Appendix 3 STROBE (Strengthening the Reporting of Observational Studies in Epidemiology) checklist.

Multimedia Appendix 4 Threshold values for abnormal measurements defined in the Minder platform.

Multimedia Appendix 5 Within-participant standard deviation for all six measurement domains; only participants with more than 7 days data are included. PwD: people with dementia.

Multimedia Appendix 6

Multimedia Appendix 7 Flowchart followed by monitoring team in response to alerts for abnormal oxygen saturation. GP: General physician.

Multimedia Appendix 8 Flowchart followed by monitoring team in response to alerts for body temperature. GP: general physician; UTI: urinary tract infection.

Multimedia Appendix 9 Participant withdrawals.

Multimedia Appendix 10 Flowchart followed by monitoring team in response to alerts for abnormal heart rate. CMT: clinical monitoring team.

Multimedia Appendix 11 Flowchart followed by monitoring team in response to alerts for abnormal blood pressure readings. BP: blood pressure; GP: general physician.

Multimedia Appendix 12

## Abbreviations

AD: Alzheimer disease
ASyn: *α*-synuclein–associated dementias
DBP: diastolic blood pressure
HR: heart rate
IoT: Internet of Things
NEWS: National Early Warning Score
SBP: systolic blood pressure
SMMSE: Standardized Mini-Mental State Examination
UTI: urinary tract infection
VD: vascular dementia

## Data Availability

The data collected during the current study are available from the corresponding author on reasonable request. The institute intends to store data in a public repository on dementiasplatform.uk in due course.
