# Supplementary material for: Remote Monitoring of Physiology in People Living With Dementia: An Observational Cohort Study
Source: JMIR Aging. 2023 Mar 9;6:e43777. doi: 10.2196/43777 (PMC10037178; doi:10.2196/43777)
Supplement: Multimedia Appendix 1 [file aging_v6i1e43777_app1.docx]

**Multimedia Appendix 1. Table S1. Threshold values for abnormal measurements in the National Early Warning System 2 (NEWS)**(32)

| Domain | Points | | | | | | |
| --- | --- | --- | --- | --- | --- | --- | --- |
|  | 3 | 2 | 1 | 0 | 1 | 2 | 3 |
| HR (bpm) | ≤40 |  | 41-50 | 51-90 | 91-110 | 111-130 | ≥131 |
| SBP (mmHg) | ≤90 | 91-100 | 101-110 | 111-219 |  |  | ≥220 |
| Temp. (C) | ≤35.0 |  | 35.1-36.0 | 36.1-38.0 | 38.1-39.0 | ≥39.1 |  |
| Sats (%) | ≤91 | 92-93 | 94-95 | ≥96 |  |  |  |
| Respiratory rate (/min) | ≤8 |  | 9-11 | 12-20 |  | 21-24 | ≥25 |
| Conscious level |  |  |  | Alert |  |  | CVPU |

Note: respiratory rate (/min) and conscious level are part of the NEWS system but were not recorded in the present study. Abbreviations: CVPU = (new) confusion, responsiveness only to voice or pain, or unresponsiveness; HR = heart rate, bpm = beats per minute, SBP = systolic blood pressure, mmHg = millimetres of mercury; ^o^C = degrees Celsius, Sats = oxygen saturation.
