# Supplementary material for: Remote Monitoring of Physiology in People Living With Dementia: An Observational Cohort Study
Source: JMIR Aging. 2023 Mar 9;6:e43777. doi: 10.2196/43777 (PMC10037178; doi:10.2196/43777)
Supplement: Multimedia Appendix 2 [file aging_v6i1e43777_app2.docx]

## Multimedia Appendix 2. Methods

### Inclusion and exclusion criteria for PwD and study partners

PwD inclusion criteria

- Living in the community
- If on anti-dementia medication (cholinesterase inhibitors and/or memantine) on a stable dose for three months prior to being recruited
- Have a study partner
- Have sufficient functional English to allow completion of the assessment instruments
- Male or female of 50 years of age and older at baseline with a confirmed diagnosis of dementia (any type) by specialist assessment

PwD exclusion criteria

- Receipt of any investigational drug within 30 days prior to consenting
- People with unstable mental state including severe depression, severe psychosis, agitation and anxiety whom their medication was changed over the last 4 weeks
- Inability to communicate verbally
- Requiring regular elective hospital admission for their physical health monitoring
- Current active suicidal ideas
- Severe sensory impairment
- Receiving treatment for terminal illness

Study partner inclusion criteria

- Willing and able to provide informed consent
- Single nominated study partner (relative/friend) who has known the person with dementia for at least 6 months and is able to attend research assessments with the person with dementia
- Have sufficient functional English to allow completion of the assessment instruments
- Aged 18 or over

Study partner exclusion criteria

- Study partners are unable to communicate verbally
- Unable to provide written informed consent
