# Supplementary material for: Remote Monitoring of Physiology in People Living With Dementia: An Observational Cohort Study
Source: JMIR Aging. 2023 Mar 9;6:e43777. doi: 10.2196/43777 (PMC10037178; doi:10.2196/43777)
Supplement: Multimedia Appendix 4 [file aging_v6i1e43777_app4.docx]

**Multimedia Appendix 4. Table S2. Threshold values for abnormal measurements defined in the Minder platform**

| Domain | Criteria |
| --- | --- |
| HR (bpm) | <55 or >99 |
| SBP (mmHg) | <81 or >159 |
| Temp. (C) | <35 or >37.6 |
| Sats (%) | <94 |
| DBP (mmHg) | <55 or >99 |
| Bodyweight (kg) | Change of ≥5% compared to the average bodyweight over the last week, month, 2 months or 3 months |

Note: DBP and bodyweight were not used in the analysis of alerts, since the focus was a comparison with the NEWS scoring system, which does not use these domains. Abbreviations: HR = heart rate, bpm = beats per minute, DBP = diastolic blood pressure; SBP = systolic blood pressure, mmHg = millimetres of mercury; ^o^C = degrees Celsius, Sats = oxygen saturation.
