# Supplementary figures and images for: Remote Monitoring of Physiology in People Living With Dementia: An Observational Cohort Study
Source: JMIR Aging. 2023 Mar 9;6:e43777. doi: 10.2196/43777 (PMC10037178; doi:10.2196/43777)

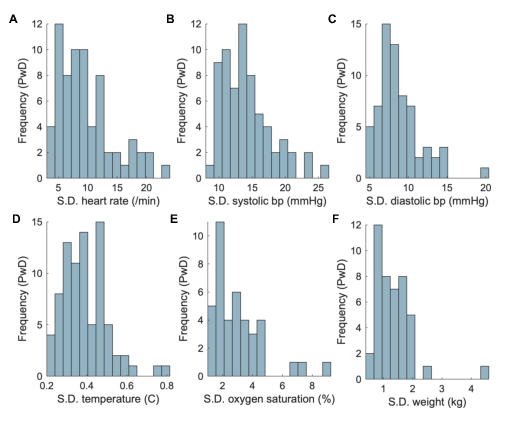

Supplement: Multimedia Appendix 5 [file aging_v6i1e43777_app5.png]

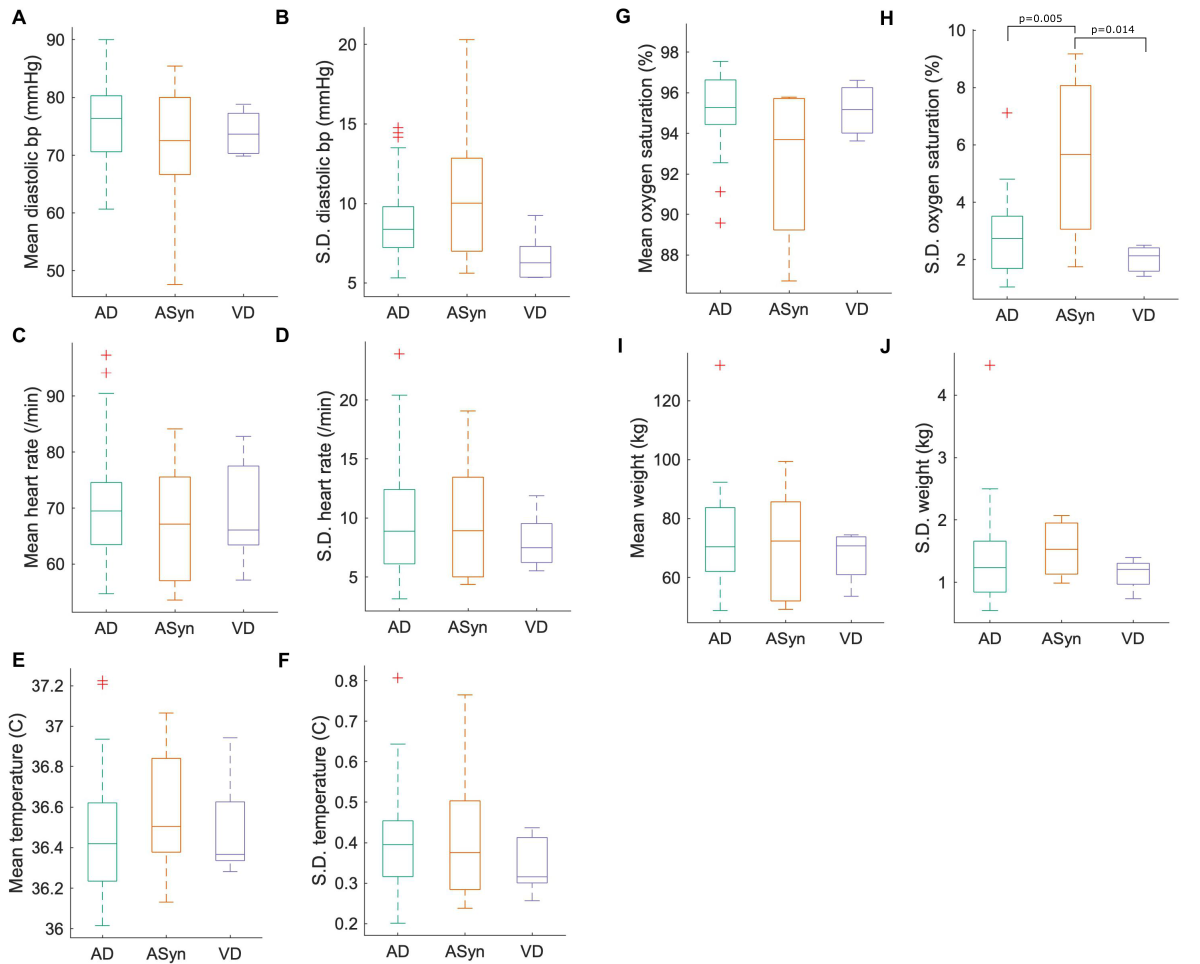

Supplement: Multimedia Appendix 6 [file aging_v6i1e43777_app6.png]

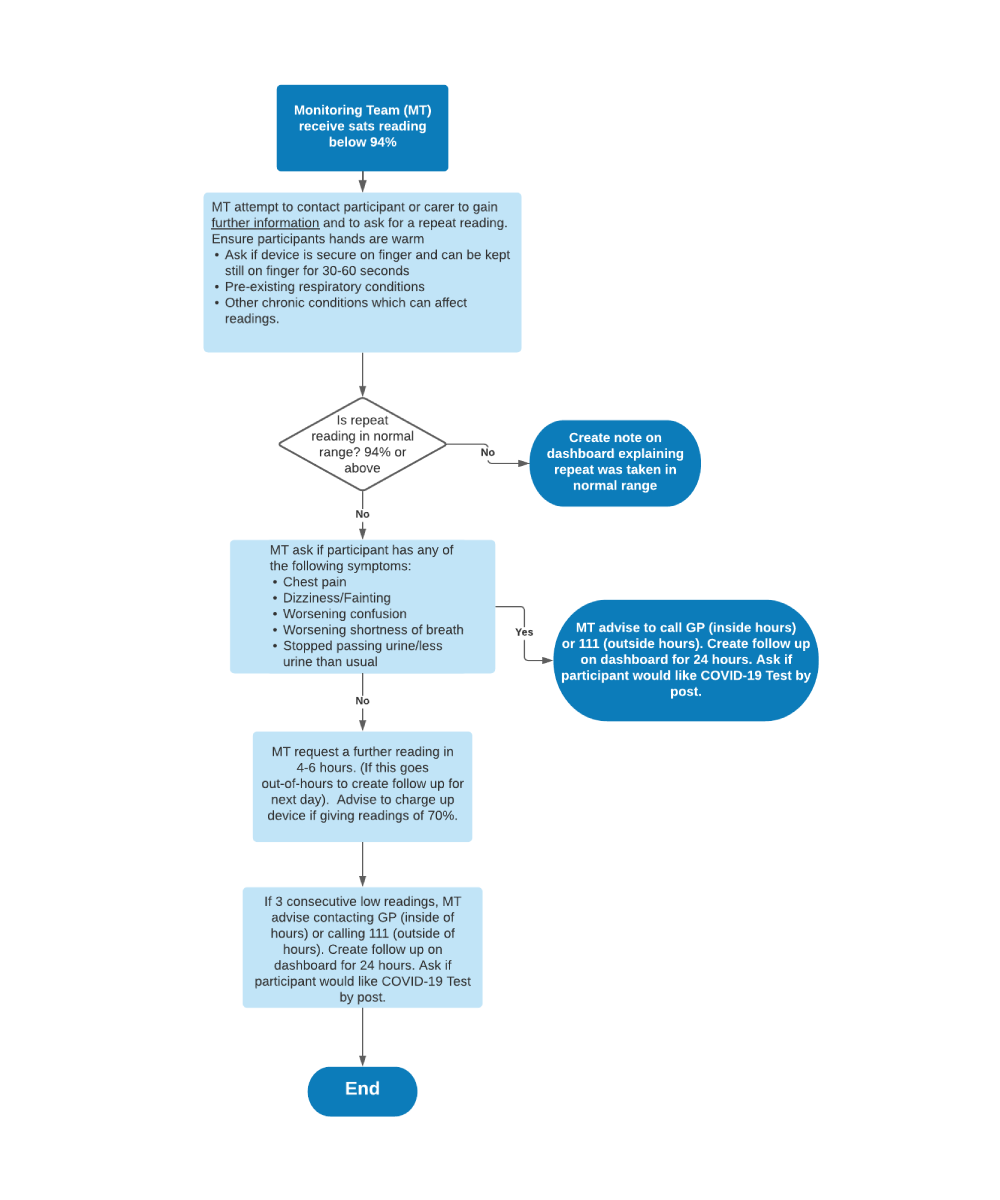

Supplement: Multimedia Appendix 7 [file aging_v6i1e43777_app7.png]

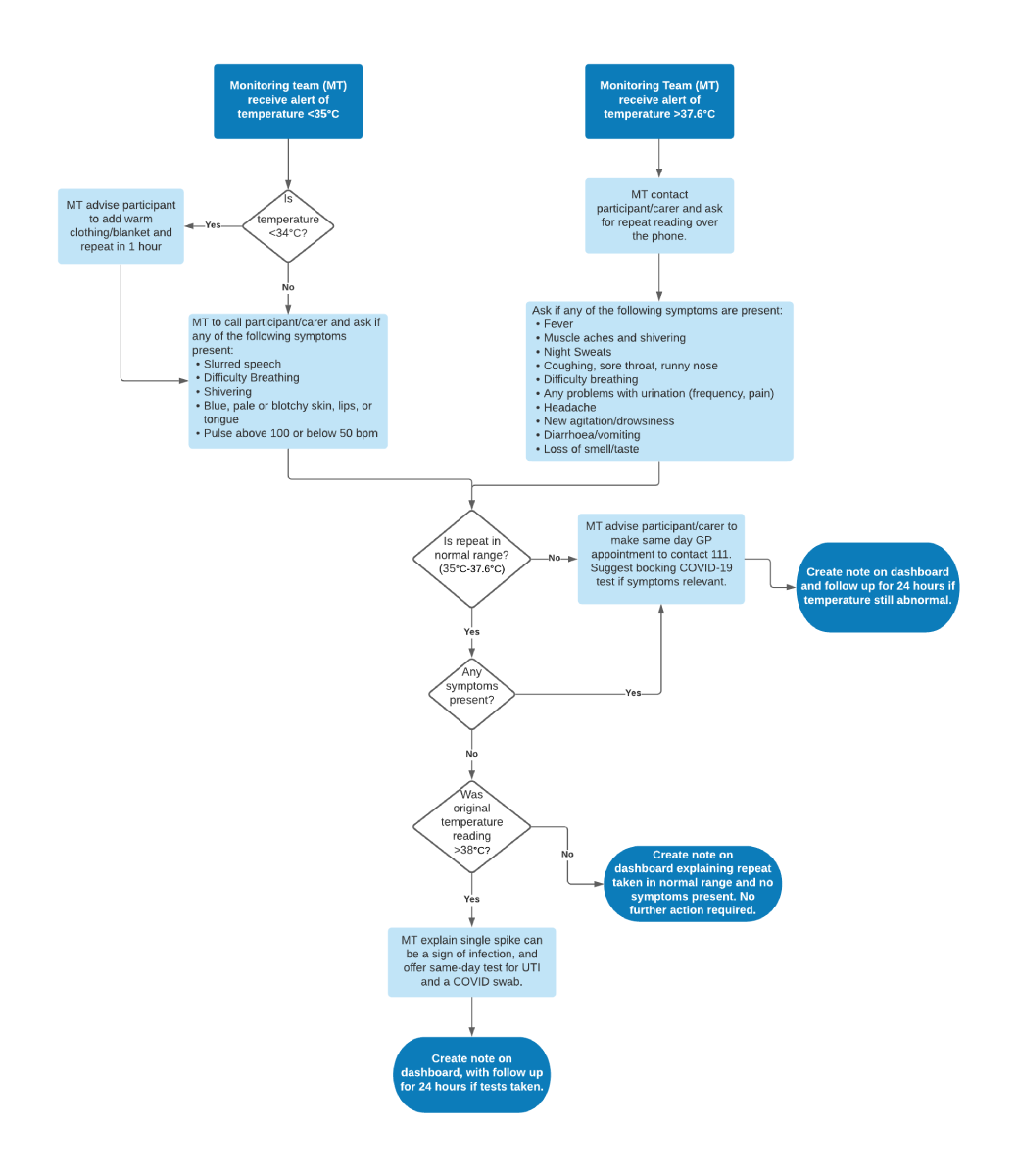

Supplement: Multimedia Appendix 8 [file aging_v6i1e43777_app8.png]

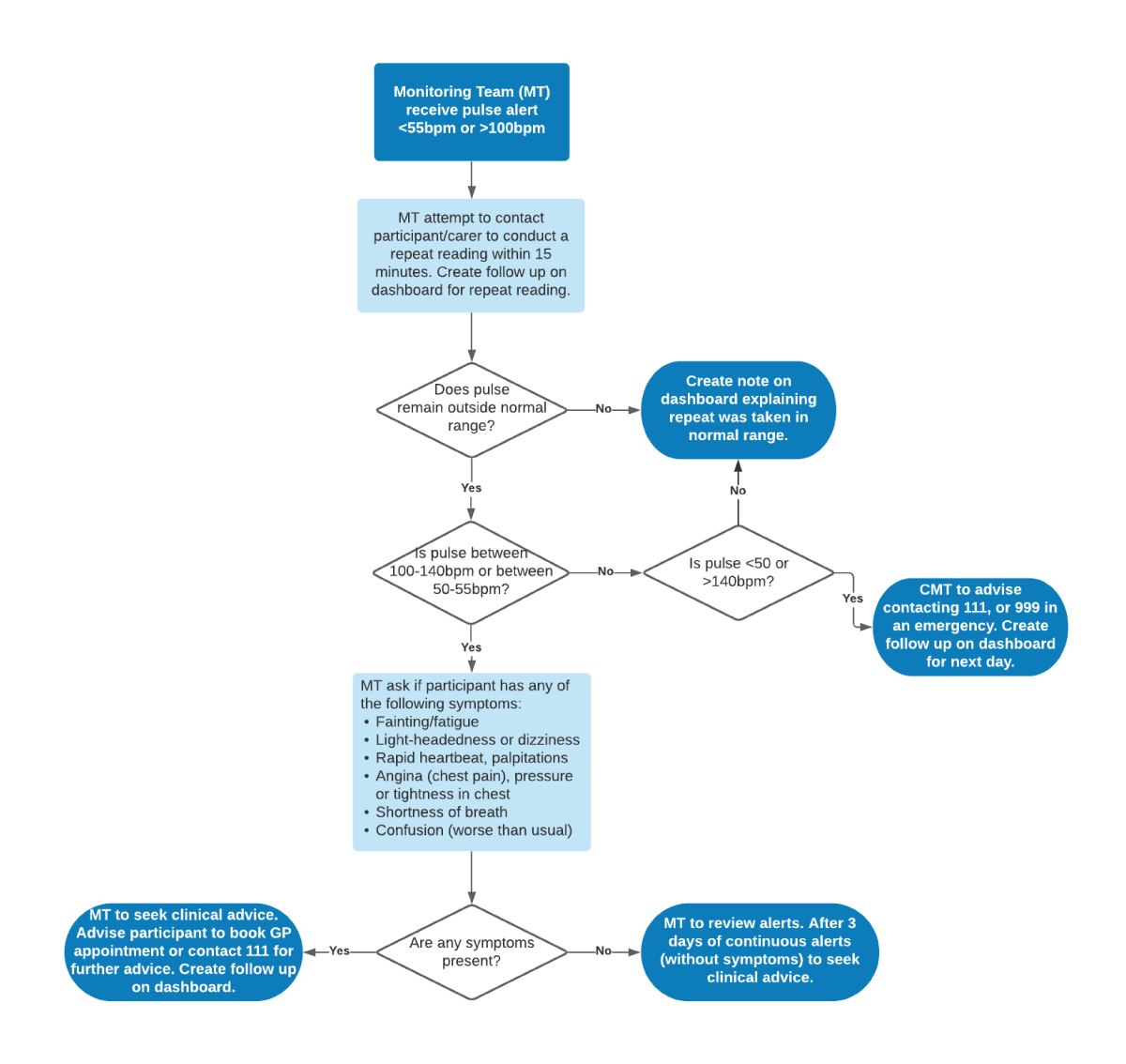

Supplement: Multimedia Appendix 10 [file aging_v6i1e43777_app10.png]

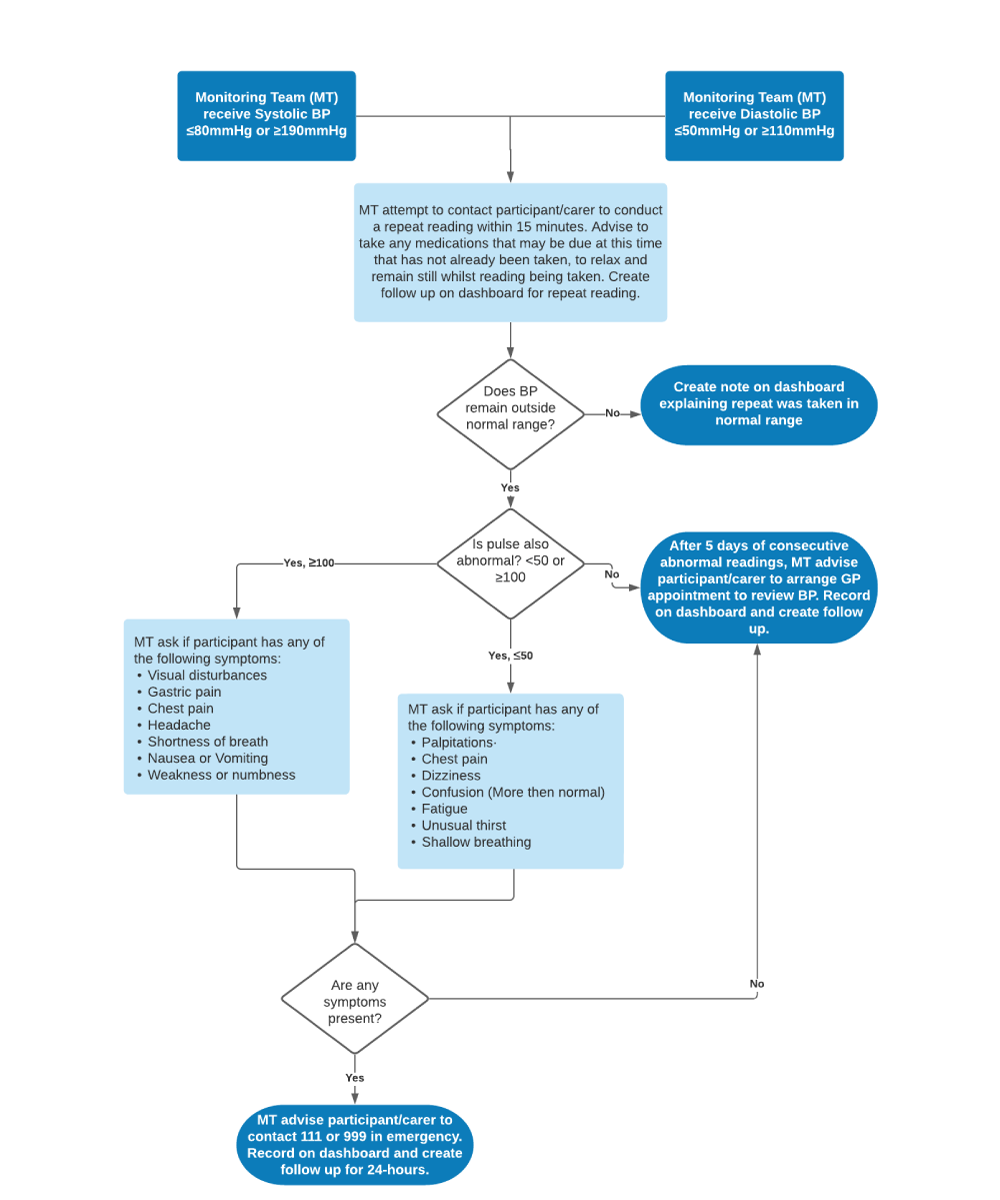

Supplement: Multimedia Appendix 11 [file aging_v6i1e43777_app11.png]
