# Supplementary material for: Remote Monitoring of Physiology in People Living With Dementia: An Observational Cohort Study
Source: JMIR Aging. 2023 Mar 9;6:e43777. doi: 10.2196/43777 (PMC10037178; doi:10.2196/43777)
Supplement: Multimedia Appendix 9 [file aging_v6i1e43777_app9.docx]

### Multimedia Appendix 9. Table S3. Participant withdrawals

37 participants withdrew from the study during the analysis period, including 5 (6.1%) that died.

| **Reason** | **Number** |
| --- | --- |
| Moved to Carehome | 10 |
| Deceased | 5 |
| Did not wish to continue | 7 |
| Moved out of area | 2 |
| Poor health | 5 |
| Excessive study burden | 4 |
| Unable to use technology | 2 |
| Lost contact | 2 |
