# Supplementary material for: Remote Monitoring of Physiology in People Living With Dementia: An Observational Cohort Study
Source: JMIR Aging. 2023 Mar 9;6:e43777. doi: 10.2196/43777 (PMC10037178; doi:10.2196/43777)
Supplement: Multimedia Appendix 12 [file aging_v6i1e43777_app12.docx]

**Multimedia Appendix 12. List of all members of UK Dementia Research Institute (UK DRI) Care Research & Technology (CR&T) Centre**

**Leadership and Management Infrastructure:**

Centre Director: Professor David Sharp

Co-Director: Professor Payam Barnaghi

Centre Manager: Danielle Wilson

Health and Social Care Lead: Sarah Daniels

Project Managers: Mara Golemme and Zaynab Ismail, Imperial College London

Group Leaders: Professor David Sharp, Professor Payam Barnaghi, Professor Paul Freemont, Dr Ravi Vaidyanathan, Professor Tim Constandinou, Imperial College London

Professor Derk-Jan Dijk, University of Surrey

**Groups: Behaviour and Cognition led by Prof David Sharp**

Michael David MD

Martina Del Giovane

Neil Graham MD PhD

Naomi Hassim

Magdalena Kolanko MD

Helen Lai

Lucia Li MD

Paresh Malhotra MD PhD

Emma Jane Mallas PhD

Greg Scott MD

Alina-Irina Serban

Eyal Soreq PhD

Tong Wu PhD

**Biosensor Hardware led by Prof Timothy Constandinou**

Alan Bannon PhD

Shlomi Haar PhD

Charalambos Hadjipanayi

Ghena Hammour

Bryan Hsieh

Adrien Rapeaux PhD

**Robotics and AI interfaces led by Dr Ravi Vaidyanathan**

Maria Lima

Maitreyee Wairagkar PhD

**Machine intelligence led by Professor Payam Barnaghi**

Nan Fletcher-Lloyd

Hamed Haddadi PhD

Valentinas Janeiko

Anna Joffe

Samaneh Kouchaki PhD

Viktor Levine

Honglin Li

Amer Marzuki

Francesca Palermo

Mark Woodbridge

Yuchen Zhao PhD

Alexander Capstick

Severin Skillman

**Point of care Diagnostics led by Professor Paul Freemont**

Loren Cameron PhD

Michael Crone PhD

Kirsten Jensen PhD

Martin Tran

Thomas Adam

**Sleep and Circadian led by Professor Derk Jan Dijk**

Anne Skeldon, PhD

Kevin Wells, PhD

Ullrich Bartsch PhD

Ciro Della Monica PhD

Kiran GR Kumar PhD

Damion Lambert

Sara Mohammadi Mahvash PhD

Thalia Rodriguez Garcia PhD

Vikki Revell PhD

Giuseppe Atzori

Lucinda Grainger

Hana Hassanin MD

James Woolley

Iris Wood-Campar

Janetta Rexha

Helix Centre – Human Centred Design led by Matthew Harrison

Sophie Horrocks

Lenny Naar

Brian Quan

**Site Investigators and Key Personnel:**

**Surrey and Borders Partnership NHS Foundation Trust (Site and Sponsor)**

Chief Investigator: Professor Ramin Nilforooshan

Research and Development Managers: Jessica True, Olga Balazikova

Research Co-ordinator: Emily Beale

Clinical Monitoring Team: Vaiva Zarombaite, Lucy Copps, Olivia Knight, Gaganpreet Bangar, Sumit Dey, Chelsea Mukonda, Jessica Hine, Luke Mallon

**Brook Green Medical Centre / Hammersmith and Fulham Site**

Principal Investigator: Dr David Wingfield

Research Nurse / Paramedic: Claire Norman

Clinical Studies Officers/Research Technicians: Anesha Patel, Ruby Lyall, Sanara Raza

Research Therapists: Naomi Hassim, Pippa Kirby

LBHF Support: Assistive Technology: John Patterson, Business Development; Mike Law,

Social Services OT: Andy Kenny.
